# The Significance of Ultrasound in Determining Whether SHPT Patients Are Sensitive to Calcitriol Treatment

**DOI:** 10.1155/2016/6193751

**Published:** 2016-02-29

**Authors:** Xing-xin Liang, Fan Li, Feng Gao, Chun-xiao Li, Xiao-hui Qiao, Jia-jie Zhang, Lian-fang Du

**Affiliations:** ^1^Department of Medical Ultrasound, Jiangwan Hospital, Hongkou, Shanghai 200434, China; ^2^Department of Medical Ultrasound, Shanghai First People's Hospital, School of Medicine, Shanghai Jiao Tong University, Shanghai 200080, China

## Abstract

This study was to explore the significance of ultrasound in determining whether the patients with secondary hyperparathyroidism (SHPT) are sensitive to calcitriol treatment. According to the decrease value of parathyroid hormone (PTH), 42 SHPT patients were divided into two groups: drug susceptible group and drug insusceptible group. These 42 SHPT patients' ultrasound images were retrospectively analyzed. The morphology, size, number, blood flow, elastic modulus, and perfusion of the parathyroid glands were correlated with drug therapeutic outcome (oral calcitriol). Most SHPT patients with drug susceptible showed volume <438.50 mm^3^ and number ≤2, with 0-1 structural and vascular patterns, associated with Relative Maximum Intensity (RIMAX) <1.59 and elastic modulus <18.8 kPa, whereas most SHPT patients with drug insusceptible showed volume ≥438.50 mm^3^ and number ≥3, with 2-3 structural and vascular patterns, associated with Relative Maximum Intensity (RIMAX) ≥1.59 and elastic modulus ≥18.8 kPa. Therefore, ultrasonography in SHPT allows an accurate definition of the morphology, size, number, blood flow, elastic modulus, and perfusion of the parathyroid glands and is useful in determining whether SHPT patients are sensitive to calcitriol treatment.

## 1. Introduction

Secondary hyperparathyroidism (SHPT) is widely prevalent in patients with chronic renal failure (CRF), characterized by parathyroid hyperplasia and elevated parathyroid hormone (PTH) [1, 2].

CRF causes hypocalcemia, phosphate retention, and 1,25-hydroxyvitamin D_3_ deficiency which stimulate the synthesis and release of PTH. Hypocalcemia and phosphate retention result in persistent overstimulation of parathyroid gland (PTG), triggering cell hypertrophy-hyperplasia, and finally result in parathyroid hyperplasia.

Treatments of SHPT include dietary phosphate restriction, administration of calcium or non calcium, containing phosphate binders and parathyroid resection. Drug therapy can reduce the synthesis and release of PTH, which appeared to be safe and well tolerated, so drug therapy is the first choice for SHPT.

Calcitriol is highly active vitamin D_3_, which is exactly the same to natural hormone produced by kidneys. It is rapidly absorbed after oral absorption and safely returns to normal level 2 to 7 days after reduction or withdrawal. It can effectively inhibit the secretion of PTH and improve bone metabolism and the clinical symptoms of patients. So calcitriol has been suggested as conventional drug therapy for SHPT, but some patients are susceptible to calcitriol while some patients are insusceptible to calcitriol. Drug insusceptibility causes PTH to rise continuously which lead to renal osteodystrophy and heterotopic calcification; anemia [3, 4]; bone disease; ischemic damage, ulcer or necrosis of skin; severe intractable pruritus; and so on, and some of its components such as hyperphosphatemia or cardiovascular are related to mortality.

Numerous studies have also shown that ultrasonography is an effective method to predict the therapeutic outcome and to plan the strategy of SHPT therapy [5–9]. Our study aimed at evaluating the relationship between ultrasonography findings including conventional ultrasound, contrast-enhanced ultrasound (CEUS) and elastography, and the drug therapeutic outcomes (whether SHPT patients are sensitive to calcitriol treatment.).

## 2. Materials and Methods

### 2.1. Patients

62 patients who underwent hemodialysis in Shanghai First People's Hospital from May 2014 to December 2014 and whose PTH level was ≥250 mg/dL were eligible for the present study (PTH level ≥ 250 mg/dL was defined as SHPT [10]). Out of 62 eligible patients, we excluded 20 patients due to not finding parathyroid hyperplasia on ultrasonography. Thus, the patient cohort consisted of 42 patients, involving 22 males and 20 females with mean age of 54.90 ± 15.89 years (range of 29–89 years). 8 patients were caused by glomerular nephritis, 14 patients were caused by chronic pyelonephritis, 10 patients were caused by hypertension, 4 patients were caused by diabetes, 1 patient was caused by renal tubulointerstitial lesions, and 5 patients were caused by polycystic kidney. Mean serum calcium level was 2.23 ± 0.11 mmol/L (range of 1.7–2.73 mmol/L). Mean serum phosphate level was 2.06 ± 0.16 mmol/L (range of 0.7–2.9 mmol/L). Mean intact PTH level was 414.48 ± 98.25 mg/dL (range of 263–1680 mg/dL). Mean creatinine serum level was 985 ± 102.42 *μ*mol/L (range of 588–1402 *μ*mol/L). The volume of PTG was calculated according to the formula *V* = (*a* × *b* × *c*) × JI/6 (where *a*, *b*, and *c* are the PTG dimensions). Serum iPTH levels were determined by immunoradiometric assay.

### 2.2. Study Design

We defined that the volume of the largest gland was ≥300 mm^3^ as parathyroid hyperplasia [11]. After finishing all the ultrasound examinations, patients would receive calcitriol treatment, 0.25 *μ*g each time, once daily, lasting for 12 successive weeks (all patients were treated only with calcitriol). And PTH should be rechecked. The decrease value of PTH was calculated before and after taking calcitriol, and patients whose PTH declined to normal level after taking calcitriol were defined as drug susceptible, while those whose PTH seemed to have no obvious change were defined as drug insusceptible.

### 2.3. Apparatus and Methods

Conventional ultrasound and elastography were performed using 4–15 MHz linear probe of Aixplorer® (Supersonic Imagine, France) and using 4–9 MHz linear probe of Sequioa 512 (SIMENS, German). All ultrasound examinations were performed by one ultrasound doctor who was engaged in the parathyroid examination for more than 5 years and image analysis was done by the other two ultrasound doctors without any clinical information. They discussed to reach a consensus when they disagreed with each other. Patients' hyperextend necks were examined in supine position, fully exposing the front of the neck. The scanning range of PTG was up to the upper jaw, lower to the upper fossa or the upper fossa, and the two sides to the carotid artery. The position and number of parathyroid glands were recorded and the length, width, and thickness of the largest gland were measured. For hyperplastic parathyroid, we needed to further observe the morphology, size, number, blood flow, elastic modulus, and perfusion of PTG and measured the elastic modulus and Relative Maximum Intensity (RIMAX) of PTG. We performed conventional ultrasound, CEUS, and elastography examination on the same gland, the largest gland of PTGs.

**Figure 1:**
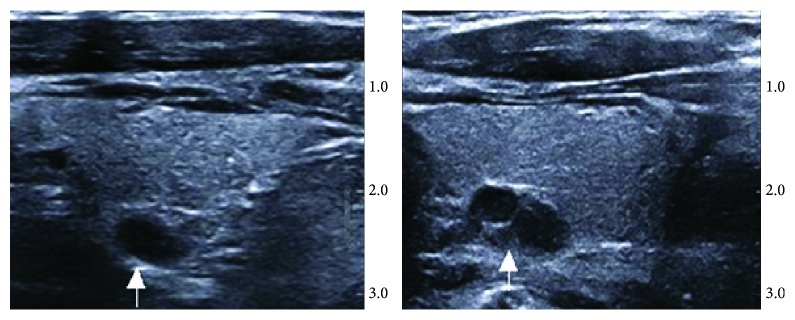
Conventional ultrasound images of parathyroid hyperplasia. In drug susceptible group, ultrasound images showed PTG with slight heterogeneousness. In drug insusceptible group, ultrasound images showed PTG with high heterogeneousness.

On conventional ultrasound, we observed the morphology, size, number, and blood flow of PTG. The morphology of PTG was classified by the flowing structural scores [10]: level 0, hypoechoic homogeneous; level 1, slightly heterogeneous; level 2, highly heterogeneous; level 3, nodular (Figure 1). The blood flow signal was classified by the following vascular scores [12]: level 0, nonvascularized pattern: an absent blood flow signal; level 1, hypovascularized pattern: very small peripheral/central blood flow signal; level 2, medium vascularized pattern: blood flow signal surrounding >30% of the PTG circumference and/or <30% of its surface; level 3, hypervascularized pattern: high peripheral and central blood flow signal >30% of the PTG surface.

Elastography was performed with Shear Wave Elastography (SWE) and started after finishing conventional ultrasound examination, using the dual frame real-time imaging, asking patient to keep normal respiration and hold his/her breath for 5–10 S. The size of the region of interest (ROI) was 3 × 3 mm (height × width) and three successful readings were recorded. Definitive elastic modulus was documented as the mean of these three readings and took kPa as a unit.

CEUS examination had been authorized by the ethics committee of Shanghai First People's Hospital, and patients signed informed consent before the inspection. CEUS was performed by a rapid 2 to 3 seconds bolus injection into antecubital vein with 3 mL sulfur hexafluoride (SonoVue, Bracco, Italy) microbubble suspension. We set MI to 0.20, used twin display mode to ensure that the location of nodule was not offset in the whole process of the contrast, recorded time and saved the image, and used SonoLiver (TomTec, German) to draw time intensity-curve (TIC). We choose the RIMAX as the evaluation index according to preexperiment. RIMAX was defined as the ratio of the maximum intensity of hyperplastic parathyroid to that of adjacent thyroid tissue.

### 2.4. Statistical Analysis

Receiver operating characteristic (ROC) analysis was performed to assess the diagnostic performance of conventional ultrasound, CEUS, and elastography in determining whether calcitriol treatment was effective or not and get the best cut-off value of morphology, size (mm^3^), number, blood flow, RMAXI, and elastic modulus (kPa).

## 3. Results

### 3.1. Conventional Ultrasound Imaging

**Figure 2:**
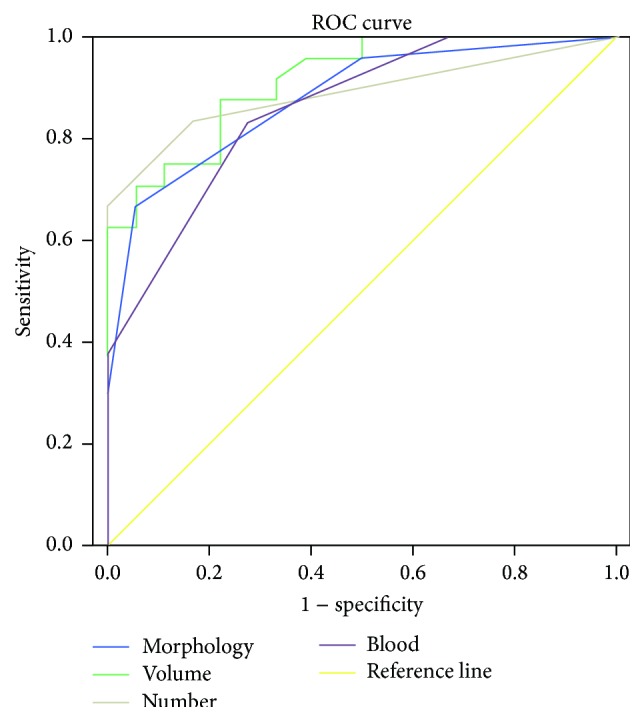
The ROC analysis of morphology, size, number, and blood flow. The ROC analysis of morphology showed that the area under the curve was 0.88 and the cut-off value was 1.5 with sensitivity of 66.7% and specificity of 94.4%. The ROC analysis of size showed that the area under the curve was 0.91 and the cut-off value was 438.50 mm^3^ with sensitivity of 87.5% and specificity of 77.8%. The ROC analysis of number of PTGs showed that the area under the curve was 0.89 and the cut-off value was 2.5 with sensitivity of 66.7% and specificity of 100%. The ROC analysis of blood flow showed that the area under the curve was 0.86 and the cut-off value was 1.5 with sensitivity of 83.3% and specificity of 72.2%.

In drug susceptible group, the mean structural score was 0.56 ± 0.62 (range of 0–2), the mean volume was 392.94 ± 74.53 mm^3^ (range of 315–538 mm^3^), the mean number was 1.17 ± 0.38 (range of 1-2), and the mean vascular score was 0.94 ± 0.80 (range of 0–2). In drug insusceptible group, the mean structural score was 1.92 ± 0.88 (range of 0–3), the mean volume was 546.04 ± 86.86 mm^3^ (range of 379–702 mm^3^), the mean number was 2.88 ± 1.12 (range of 1–4), and the mean vascular score was 2.21 ± 0.72 (range of 1–3). ROC analysis of morphology showed that the area under the curve was 0.88 and the cut-off value was 1.5 with sensitivity of 66.7% and specificity of 94.4%. ROC analysis of volume showed that the area under the curve was 0.91 and the cut off value was 438.50 mm^3^ with sensitivity of 87.5% and specificity of 77.8%. ROC analysis of number showed that the area under the curve was 0.89 and the cut off value was 2.5 with sensitivity of 66.7% and specificity of 100%. ROC analysis of blood flow showed that the area under the curve was 0.86 and the cut-off value was 1.5 with sensitivity of 83.3% and specificity of 72.2% (Figure 2).

### 3.2. CEUS Imaging

**Figure 3:**
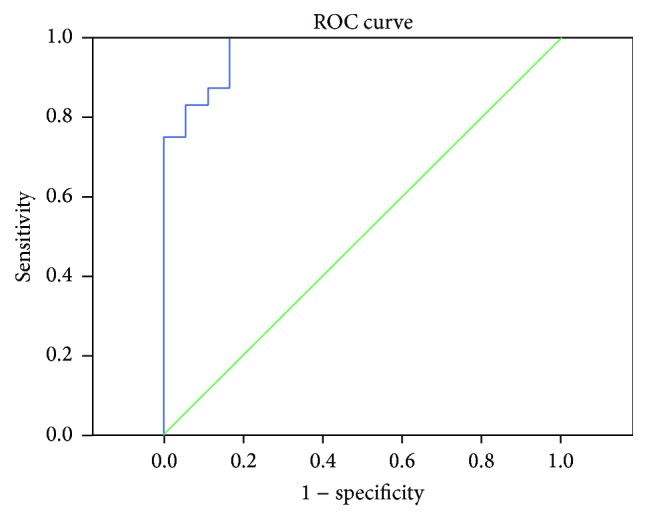
The ROC analysis of RMAXI. The ROC analysis of RMAXI showed that the area under the curve was 0.97 and the cut-off value was 1.59 with sensitivity of 100% and specificity of 83.3%.

We made TIC analysis on patients' CEUS images. Their mean RMAXI of drug susceptible group was 0.74 ± 0.68 (range of 0.18–1.91). Their mean RMAXI of drug insusceptible group was 2.25 ± 0.48 (range of 1.59–3.21). ROC analysis showed that the area under the curve was 0.97 and the cut-off value was 1.59 with sensitivity of 100% and specificity of 83.3% (Figure 3).

### 3.3. Elastography Imaging

**Figure 4:**
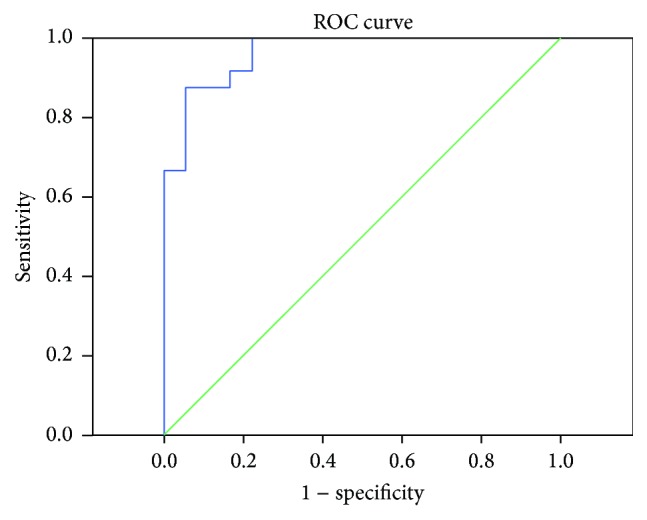
The ROC analysis of elastic modulus. The ROC analysis of elastic modulus showed that the area under the curve was 0.96 and the cut-off value was 18.8 kPa with sensitivity of 87.5% and specificity of 94.4%.

The mean elastic modulus of ROI of drug susceptible group was 9.87 ± 4.75 kPa (range of 2.9–19.8 kPa). Their mean elastic modulus of ROI of drug insusceptible group was 20.56 ± 3.55 kPa (range of 11.1–25.8 kPa). ROC analysis showed that the area under the curve was 0.96 and the cut-off value was 18.8 kPa with sensitivity of 87.5% and specificity of 94.4% (Figure 4).

## 4. Discussion

Because of its inexpensive, noninvasive, and its sufficient specificity to assess not only localization, but also the size, shape, and type of parathyroid hyperplasia, ultrasonography is a preferred examination method for parathyroid hyperplasia. Meola et al. said [13] that ultrasound can evaluate the effect of therapy which is changing the natural history of SHPT and the evaluation of morphological and vascular changes of hyperplastic parathyroids is useful to guide percutaneous ethanol injection therapy and to support clinical, pharmacological, and surgical strategies. The therapies for SHPT include drug therapy and surgical therapy. Because patients need to take calcium supplementation for a long time after operation, which might last for more than 1 year or for lifetime [14] and the recurrence rate of hyperparathyroidism 6 months after operation might reach 30.8% [15], drug therapy is the first choice for SHPT patient and nonselective vitamin D receptor activators (VDRA), such as calcitriol, have been successfully used in the treatment of SHPT [16]. Although calcitriol has beneficial effects on the control of serum PTH levels, some patients are calcitriol-resistant. So the key to treat SHPT was to determine whether calcitriol treatment is effective. Negri and Brandemburg said [17] that ultrasound findings can help clinic to early identify those patients who will not respond appropriately to calcitriol.

What is the advantage of using ultrasound to identify drug susceptible or drug insusceptible? Reichel [18] said that, in patients with diffuse hyperplasia, treatment by calcitriol is recommended; Katoh et al. [19] said that, in patients with nodular hyperplasia, treatments with calcitriol most probably will not be effective. Based on our study, we use ultrasonography to clearly detect morphology, size, number, and blood flow of the PTGs and carry out semiquantitative analysis, which can be used to assess the therapeutic response of SHPT. Furthermore, we used CEUS and elastography to further quantitatively analyze the difference of the RIMAX and elastic modulus between drug susceptible group and drug insusceptible group. Low mechanical index CEUS can dynamically observe the perfusion status and changes of microvessel in real time. As a novel elastography technique, SWE possesses the quality of quantitative analysis, real-time, and high spatial resolution. Our study showed that CEUS and elastography have higher accuracy in determining whether SHPT patients are sensitive to calcitriol treatment than conventional ultrasound.

Conventional ultrasound examination has high accuracy in determining whether calcitriol treatment is effective. Most SHPT patients with drug susceptible showed volume <438.50 mm^3^ and number ≤2, with 0-1 structural and vascular patterns, whereas most SHPT patients with drug insusceptible showed volume ≥438.50 mm^3^ and number ≥3, with 2-3 structural and vascular patterns.

CEUS examination has high accuracy in determining whether calcitriol treatment is effective. Most SHPT patients with drug susceptible showed RIMAX <1.59, whereas most SHPT patients with drug insusceptible showed RIMAX ≥1.59.

Elastography examination has high accuracy in determining whether calcitriol treatment is effective. Most SHPT patients with drug susceptible showed elastic modulus <18.8 kPa, whereas most SHPT patients with drug insusceptible showed elastic modulus ≥18.8 kPa.

## 5. Conclusions

Through quantitative or semiquantitative analysis, the present study suggests that ultrasonography in SHPT allows an accurate definition of the morphology, size, number, blood flow, elastic modulus, and perfusion of the PTGs which is useful in determining whether SHPT patients are sensitive to calcitriol treatment and may help clinicians to plan the strategy of SHPT therapy. Further studies are needed to evaluate if ultrasonography is also useful in predicting the effects of other therapeutic methods for SHPT.
